# Supplementary material for: Identification of Freezing-Responsive microRNAs and Their Targets in Chinese Jujube by Small RNA and Degradome Sequencing
Source: Int J Mol Sci. 2026 Sep 4;27(17):7912. doi: 10.3390/ijms27177912 (PMC13566890; doi:10.3390/ijms27177912)
Supplement: Supplementary file 1 [file ijms-27-07912-s001.zip › Supplementary Material S2/novel_miR_107.pdf]

Provisional ID	:	novel_miR_107
Score total	:	1033.8
Score for star read(s)	:	3.9
Score for read counts	:	1032
Score for mfe	:	0
Score for randfold	:	-2.2
Score for cons. seed	:	
Total read count	:	2036
Mature read count	:	1998
Loop read count	:	37
Star read count	:	1

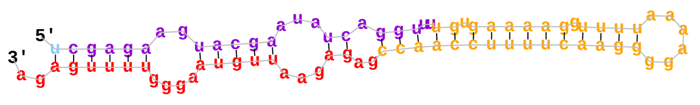

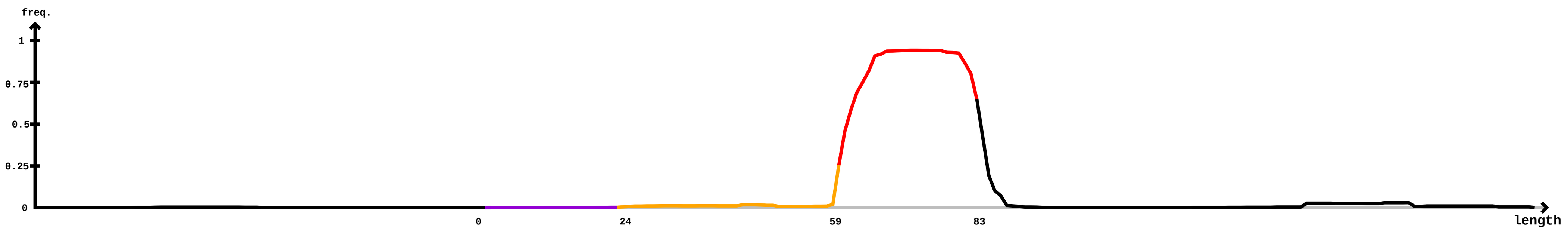

Star Mature

```
5'- uacauuuuaauuauauauuugggguauuuauuuauuuauuuguuguuuguuaaauuauuugguuccuugguuuucgagaaguacgaauaucagguuuugugaaaagguuuuaaaaggggaacuuuuccaaccgagagaauuguaagguuuugagagaaauuauuauuuccaauuaauuauuaucuaucugcuuauuaccgugguauuauauuguauaguagauaggucacucacugagaugauuagcau -3'   obs
    uacauuuuaauuauauauuugggguauuuauuuauuuauuuguuguuuguuaaauuauuugguuccuugguuuucgagaaguacgaauaucagguuuugugaaaagguuuuaaaaggggaacuuuuccaaccgagagaauuguaagguuuugagagaaauuauuauuuccaauuaauuauuaucuaucugcuuauuaccgugguauuauauuguauaguagauaggucacucacugagaugauuagcau        exp
    ......(((((((.....((((..........................(((((.((((.....(((((((((((..(((((...((.(((..((.((((((.((((......))))))))))))))))..))...)))))....))))))))...))))......)))).)))))...((((((((((...(((.(((......))).))).)))))))))))..)))).....))))))))...
```

read	reads	mm	sample
auauuugggguauuuauuuauC	2	1	LCK-3
uauuugggguauuuauuu	1	0	LT-2
uugggguauuuauuuauu	2	0	LCK-3
ugggguauuuauuuauuua	1	0	LT-3
uuguuaaauuauuugguCccuugg	1	1	LT-1
cgGgaaguacgaauaucagguuu	1	1	LT-2
cgaauaucGgguuuugugaaaag	1	1	JCK-3
gguuuugugaaaagguuuuU	1	1	LT-3
uuuugugaaaagguuuuaaaagggg	1	0	LT-1
ugugaaaagguuuuaaaagggga	1	0	LT-1
ugugaaaagguuuuaaaaggggG	1	1	LT-1
ugugaaaagguuuuaaaaggggaac	7	0	LCK-3
gugaaaagguuuuaaaagggga	2	0	LT-2
gugaaaagguuuuaaaaggggaac	2	0	JCK-2
gugaaaagguuuuaaaaggggaac	1	0	JCK-3
gugaaaagguuuuaaaaggggaac	3	0	JCK-1
gaaaagguuuuGaaagggg	2	1	JCK-2
aaGgguuuuaaaaggggaac	1	1	LT-3
aagguuuuaaaaggggaac	1	0	LT-3
uuaaaCggggaacuuuuc	1	1	LT-1
gggaacuuuuccaaccgagC	14	1	JT-1
uccaaccgagagaauuguaag	1	0	LCK-3
aaccgagagaaCuguaaggg	1	1	JCK-1
aaccgagagaaCuguaaggg	1	1	JCK-2
aaccgagagaauuguaagAgu	1	1	LT-2
ccgagagaauuguaaggguuuug	1	0	JCK-3
ccgagagaauuguaaggguuuug	1	0	JCK-2
cgagUgaauuguaaggguuuu	1	1	LT-1
cgagagaauuguaaggguuuuga	1	0	JCK-1
cgagagaauuguaaggguuuuga	3	0	JCK-3
cAagagaauuguaaggguuuuga	1	1	JCK-3
cAagagaauuguaaggguuuugag	1	1	JCK-3
cgagagaauuguaaggguuuugag	4	0	JCK-2

Star **Mature**

uacauuuuaauuauauauuugggguauuuauuuauuuauuuguuguuuguuaaauuauuugguuccuugguuuucgagaaguacgaauaucagguuuugugaaaagguuuuaaaaggggaacuuuuccaaccgagagaauuguaaggguuuugagagaaauuauuauuuccaauuaauuauuaucuaucugcuuauuaccgugguauuauauuguauaguagauaggucacucacugagaugauuagcau

cgagagaauuguaaggguuuugag	5	0	JCK-1
cgagagaauuguaaggguuuugag	1	0	LT-2
cgagagaauuguaaggguuuugag	1	0	LT-3
cgagagaauuguaaggguuuugag	4	0	JCK-3
gagagaauuguaaggguA	1	1	JCK-3
gagagaauuguaaggguA	1	1	JCK-1
gagagaauuguaaggguA	8	1	LT-2
gagagaauuguaaggguu	7	0	LCK-3
gagagaauuguaaggguu	5	0	LT-3
gagagaauuguaaggguu	1	0	LT-2
gagagaauuguaaggguuu	1	0	LT-3
gagagaauuguaaggguuuu	1	0	LT-1
gagagaauuguaaggguuuu	1	0	LT-3
gagagaauuguaaggguuuu	2	0	LT-2
gagagaauuguaaggguuuug	3	0	JCK-1
gagagaauuguaaggguuuug	15	0	LCK-3
gagaUaauuguaaggguuuug	1	1	JCK-3
gagagaauuguaaggguuuug	5	0	LT-3
Uagagaauuguaaggguuuug	2	1	JCK-3
Uagagaauuguaaggguuuug	1	1	JCK-2
gagagaauuguaaggguuuug	14	0	LCK-1
gagagaauuguaaggguuuug	5	0	LT-2
gagagaauuguaaggguuuug	4	0	JCK-2
gagagaauuguaaggguuuug	5	0	LT-1
gagagaauuguaaggguuuug	17	0	LCK-2
gagagaauuguaaggguuuuUa	1	1	LCK-3
gagagaauuguaaggguuuuga	2	0	LT-3
gagagaauuguaagUguuuuga	2	1	LT-2
gagagaauuguaaggguuuuga	2	0	LT-1
gagagaauuguaaggguuuuga	14	0	LCK-3
gagagaauuguaaggguuuugag	15	0	LT-2
Aagagaauuguaaggguuuugag	1	1	JT-3
Cagagaauuguaaggguuuugag	1	1	JCK-2
gagagaauuguaaggguuuugag	7	0	LT-3
gagagaauuguaaggguuuugag	5	0	JT-3
gagagaauuguaagAguuuugag	1	1	LT-2
Aagagaauuguaaggguuuugag	1	1	JT-2
gagagaauuguaaggguuuugag	18	0	LCK-3
gagagaauuguaaggguuuugag	16	0	JCK-3
gagagaauuguaaggguuuugag	6	0	JT-2
gagagaauuguaaggguuuugag	7	0	LT-1
gagagaauuguaaUgguuuugag	1	1	LCK-3
gagagaauuguaagAguuuugag	6	1	LCK-3
gagagaauuguaaggguuuugag	15	0	JCK-2
gagagaaCuguaaggguuuugag	1	1	LT-1
gagagaauuguaaggguuuugag	16	0	JCK-1
gagagaauuguaaggguuuugaga	100	0	JCK-2
gagagaauuguaGggguuuugaga	1	1	JCK-1
gCgagaauuguaaggguuuugaga	1	1	JCK-1
gagagaauuguaaggguuuugaga	2	0	JT-3
gagaUaauuguaaggguuuugaga	2	1	JCK-2
gagagaauuguaaggguuuugaga	3	0	JT-2
gagagaauuguaaggguuuugaga	3	0	LCK-3
gagagaauuguaagggCuuugaga	2	1	JCK-1
gaAagaauuguaaggguuuugaga	1	1	JCK-3
gagaUaauuguaaggguuuugaga	1	1	JCK-1
gGgagaauuguaaggguuuugaga	1	1	JCK-2
Aagagaauuguaaggguuuugaga	1	1	JCK-2
gagagaauuguaagggCuuugaga	1	1	JCK-2
gagagaauuguaaggguuuugaga	58	0	JCK-3
Aagagaauuguaaggguuuugaga	1	1	JCK-3
gagagaauuguaaggguuuugaga	7	0	LT-1
gagagaauuguaaggguuuugaga	4	0	LT-3
gagagaauuguaaggguuuugaga	10	0	LT-2
gagagaauuguaagggCuuugaga	2	1	JCK-3
gagagaauuguaaggguuuugaga	58	0	JCK-1
gagagaCuuguaaggguuuugaga	1	1	JCK-3
gagagaauuguaaggguuuugagag	2	0	JCK-2
gagagaauuguaaggguuuugagag	1	0	LT-2
gagagaauuguaaggguuuugagag	2	0	JCK-3

Star (purple) — **Mature** (red)

uacauuuuaauuauauauuugggguauuuauuuauuuauuuguuguuuguuaaauuauuuggguuccuugguuuucgagaaguacgaauaucagguuuugugaaaagguuuuaaaaggggaacuuuuccaaccgagagaauuguaaggguuuugagagaaauuauuauuuccaauuaauuauuaucuaucugcuuauuaccgugguauuauauuguauaguagauaggucacucacugagaugauuagcau

gagagaauuguaaggguuuugagaga	1	0	LT-1
gagagaauuguaaggguuuugagagaa	1	0	LT-1
agagaauuguaaggguuu	1	0	LT-3
agagaauuguaaggguuuu	1	0	LT-1
agagaauuguaaggguuuug	6	0	LT-2
agagaauuguaaggguuuug	3	0	LT-3
agagaauuguaaggguuuug	3	0	JCK-1
agCgaauuguaaggguuuug	1	1	LT-1
agagaauuguaaggguuuug	1	0	JCK-2
agagaauuguaaggguuuug	3	0	JCK-3
agagaauuguaaggguuuug	1	0	LT-1
agagaauuguaaggguuAug	1	1	JCK-1
agagaauuguaaggguuuug	10	0	LCK-3
agagaauuguaaggguuuuga	3	0	LCK-1
agagaauuguaaggguuuuga	4	0	JT-2
agagaauuguaaggguuuuga	1	0	LT-1
agagaauuguaaggguuuuga	2	0	LT-3
agagaauuguGaggguuuuga	1	1	LT-1
agagaauuguaaggguuuuga	5	0	LCK-2
agagaauuguaaggguuuuga	3	0	LCK-3
agagaauuguaaggguuuuga	1	0	JT-3
agagaauuguaaggguuuugag	6	0	LT-3
agagaaGuguaaggguuuugag	1	1	LT-3
agagaauuguaaggguuuugag	18	0	LT-2
agCgaauuguaaggguuuugag	2	1	LT-2
agagaauuguaaggguuuugag	15	0	JT-1
agagaauuguaaggguuuugag	7	0	LCK-3
agagaCuuguaaggguuuugag	1	1	LT-2
agagaauuguaaggguuuugag	13	0	JCK-1
agagaauuguaaggguuuugag	11	0	JCK-2
agagaauuguaaggguuuugag	3	0	LT-1
agagCauuguaaggguuuugag	1	1	LCK-3
agagaauuguaaggguuuugag	10	0	JCK-3
agagaauuguaaggguuuugaga	15	0	LT-2
agagaauuguaaggguuuugaga	42	0	JCK-1
agagaauuguCaggguuuugaga	1	1	LCK-2
agagaauuguCaggguuuugaga	2	1	LT-2
agagaauuguaaggguuuugaga	8	0	LT-1
agagaauuguaaggguuuugaga	17	0	LCK-1
agagaauuguaaggguuuugaga	48	0	JCK-2
agagaauuguaaggguuuugaga	4	0	LT-3
agagaauuguaCggguuuugaga	1	1	LT-3
agCgaauuguaaggguuuugaga	3	1	JCK-3
agagaauuguaaggguuuugaga	2	0	JT-3
agagaauuguaaggguuuugaga	33	0	JCK-3
agagaauuguaaggguuuugaga	6	0	LCK-3
agagaauuguaaggguuuugaga	15	0	LCK-2
aAagaauuguaaggguuuugaga	1	1	JCK-1
agCgaauuguaaggguuuugaga	1	1	JCK-1
agagGauuguaaggguuuugaga	1	1	JCK-3
agCgaauuguaaggguuuugaga	1	1	JCK-2
agagaauuguaaggguuuugaga	2	0	JT-1
agagaauuguaaggguuuugagag	13	0	JCK-3
agagaauuguaagAguuuugagag	3	1	JCK-2
agagaauuguaaggguuuugagaC	1	1	JCK-2
agagaauuguaaggguuuugagag	3	0	LT-3
agagaauuguaaggguuuugagaC	2	1	JCK-3
agagaauuguaaggguuuugagag	5	0	LT-1
agagaauuguaaggguuuugagag	15	0	JCK-1
agagaauuguaaggguuuugagag	6	0	LT-2
agagaauuguaaggguuuugagag	1	0	LCK-3
agagaauuguaaggguuuugagag	19	0	JCK-2
agagaauuguaagAguuuugagag	2	1	JCK-1
agagaauuguaaggguuuugagaga	5	0	LCK-3
agagaauuguaaggguuuugagaga	1	0	LT-3
agagaauuguaaggguuuugagaga	1	0	JCK-1
agagaauuguaaggguuuugagaga	1	0	JCK-2
agagaauuguaaggguuuugagaga	4	0	LT-2
agagaauuguaaggguuuugagagaa	4	0	LCK-3
agCgaauuguaaggguuuugagagaa	3	1	LCK-3

```
                                                                                Star                                                    Mature
uacauuuuaauuauauauuugggguauuuauuuauuuauuuguuguuuguuaaauuauuugguuccuugguuuucgagaaguacgaauaucagguuuugugaaaagguuuuaaaaggggaacuuuuccaaccgagagaauuguaaggguuuugagagaaauuauuauuuccaauuaauuauuaucuaucugcuuauuaccgugguauuauauuguauaguagauaggucacucacugagaugauuagcau
.....................................................................................................................................agagaauuguaaggguuuugagagaCa..........................................................................................   3   1   LT-1
.....................................................................................................................................agagaauuguaaggguuuugagagaCa..........................................................................................   2   1   LT-2
......................................................................................................................................gagaauuguaaggguuuu..................................................................................................   1   0   LT-1
......................................................................................................................................gagaauuguaaggguuuu..................................................................................................   1   0   LT-3
......................................................................................................................................gagaauuguaaggguuuug.................................................................................................   1   0   LT-3
......................................................................................................................................gagaauuguaaggguuuug.................................................................................................   1   0   LT-2
......................................................................................................................................gagCauuguaaggguuuug.................................................................................................   1   1   LT-2
......................................................................................................................................gagaauuguaaggguuuug.................................................................................................   13  0   JT-1
......................................................................................................................................gagaauuguaaggguuuug.................................................................................................   1   0   JCK-2
......................................................................................................................................gagaauuguaaggguuuuga................................................................................................   4   0   LT-3
......................................................................................................................................gagaauuguaaggguuuuga................................................................................................   2   0   JCK-3
......................................................................................................................................gagaauuguaaggguuuuga................................................................................................   24  0   LCK-1
......................................................................................................................................gCgaauuguaaggguuuuga................................................................................................   1   1   LCK-2
......................................................................................................................................gagaauuguaaggNuuuuga................................................................................................   1   1   LCK-1
......................................................................................................................................gagaauuguaaggguuuuga................................................................................................   24  0   LCK-2
......................................................................................................................................gagaauuguaaggguuuuga................................................................................................   1   0   LT-1
......................................................................................................................................gagaauuguaaggguuuugag...............................................................................................   1   0   JCK-1
......................................................................................................................................gagaauuguaaggguuuugag...............................................................................................   4   0   LT-3
......................................................................................................................................Aagaauuguaaggguuuugag...............................................................................................   2   1   JCK-1
......................................................................................................................................gagaauuguaaggguuuugag...............................................................................................   7   0   LCK-3
......................................................................................................................................gagaauuguaaggguuuugag...............................................................................................   3   0   JCK-2
......................................................................................................................................gagaauuguaaggguuuugag...............................................................................................   1   0   LT-1
......................................................................................................................................gagaauuguaaggguuuugag...............................................................................................   9   0   LT-2
......................................................................................................................................gagaauuguaaggguuuugag...............................................................................................   1   0   JCK-3
......................................................................................................................................gagaauuguaaggguuuugaga..............................................................................................   3   0   JCK-2
......................................................................................................................................gagaauuguGaggguuuugaga..............................................................................................   2   1   JCK-1
......................................................................................................................................gagaauuguaaggguuuugaga..............................................................................................   1   0   JCK-3
......................................................................................................................................gagaauuguaaggguuuugaga..............................................................................................   4   0   LT-3
......................................................................................................................................gagaauuguGaggguuuugaga..............................................................................................   1   1   JCK-2
......................................................................................................................................gagaauuguaaggguuuugaga..............................................................................................   8   0   LT-2
......................................................................................................................................gagaauuguaaggguuuugaga..............................................................................................   5   0   JCK-1
......................................................................................................................................Aagaauuguaaggguuuugaga..............................................................................................   1   1   LCK-3
......................................................................................................................................gagaCuuguaaggguuuugaga..............................................................................................   1   1   LT-2
......................................................................................................................................gagaauuguaaggguuuugaga..............................................................................................   5   0   LT-1
......................................................................................................................................gagaauuguaaggCuuuugaga..............................................................................................   2   1   JCK-2
......................................................................................................................................gagaauuguaaggguuuugaga..............................................................................................   6   0   JT-1
......................................................................................................................................gagaauuguaaggguuuugaga..............................................................................................   18  0   LCK-3
......................................................................................................................................gagGauuguaaggguuuugagag.............................................................................................   1   1   JCK-2
......................................................................................................................................gagaauuguaaggguuuugagag.............................................................................................   8   0   LCK-3
......................................................................................................................................gagaauuguaaggguuuugagag.............................................................................................   3   0   LT-2
......................................................................................................................................gagaauuguaaggguuuugagag.............................................................................................   2   0   LT-1
......................................................................................................................................gagGauuguaaggguuuugagag.............................................................................................   2   1   JCK-1
......................................................................................................................................gagaauuguaaggguuuugagag.............................................................................................   2   0   JCK-1
......................................................................................................................................gagaauuguaaggguuuugagag.............................................................................................   8   0   JCK-2
......................................................................................................................................Aagaauuguaaggguuuugagag.............................................................................................   1   1   JCK-1
......................................................................................................................................gagaauuguaaggguuuugagag.............................................................................................   7   0   JCK-3
......................................................................................................................................gagaCuuguaaggguuuugagag.............................................................................................   1   1   JCK-1
......................................................................................................................................gagaauuguaGggguuuugagaga............................................................................................   2   1   JCK-2
......................................................................................................................................gagaauuguaaggguuuugagaga............................................................................................   7   0   LCK-3
......................................................................................................................................gagaauuguaagggCuuugagaga............................................................................................   2   1   JCK-2
......................................................................................................................................gagaauuguaGggguuuugagaga............................................................................................   1   1   JCK-3
......................................................................................................................................Aagaauuguaaggguuuugagaga............................................................................................   3   1   JCK-1
......................................................................................................................................gagaauuguaaggguuuugagaga............................................................................................   14  0   JCK-3
......................................................................................................................................gagaauuguaaggguuuugagaga............................................................................................   19  0   JCK-2
......................................................................................................................................gagaauuguaaggguuuugaAaga............................................................................................   3   1   LT-2
......................................................................................................................................gagaauuguaaggguuuugagaga............................................................................................   8   0   JCK-1
......................................................................................................................................Aagaauuguaaggguuuugagaga............................................................................................   2   1   JCK-3
......................................................................................................................................gagaauuguaagggCuuugagaga............................................................................................   3   1   JCK-3
......................................................................................................................................gagaauuguaaggguuuugagaga............................................................................................   4   0   LT-1
......................................................................................................................................gagaauuguaaggguuuugagaga............................................................................................   3   0   LT-2
......................................................................................................................................gagaauuguaGggguuuugagaga............................................................................................   1   1   JCK-1
......................................................................................................................................gagaauuguaagggCuuugagaga............................................................................................   1   1   JCK-1
......................................................................................................................................gagaauuguaaggguuuugagagaa...........................................................................................   1   0   LCK-3
......................................................................................................................................gagaauuguaaggguuuugagagaa...........................................................................................   1   0   LT-3
.......................................................................................................................................Cgaauuguaaggguuuug.................................................................................................   2   1   LT-1
.......................................................................................................................................agaauuguaaggguuuug.................................................................................................   4   0   LT-3
.......................................................................................................................................agaauuguaaggguuuug.................................................................................................   1   0   LT-1
.......................................................................................................................................agaauuguaaggguuuuga................................................................................................   1   0   LCK-3
.......................................................................................................................................agaauuguaaggguuuuga................................................................................................   1   0   LT-3
.......................................................................................................................................agaauuguaaggguuuuga................................................................................................   1   0   LT-2
```

Star Mature

uacauuuuaauuauauauuugggguauuuauuuauuuauuuguuguuuguuaaauuauuugguuccuugguuuucgagaaguacgaauaucagguuuugugaaaagguuuuaaaaggggaacuuuuccaaccgagagaauuguaaggguuuugagagaaauuauuauuuccaauuaauuauuaucuaucugcuuauuaccgugguauuauauuguauaguagauaggucacucacugagaugauuagcau			
....agaauuguaaggguuuugag...	8	0	LT-2
....Cgaauuguaaggguuuugag...	4	1	LT-2
....agaauuguaaggguuuugag...	3	0	LT-1
....agaauuguaaggguuuugag...	7	0	LT-3
....agaauuguaaggguuuugag...	11	0	LCK-3
....agaauuguaaggguuuugaA...	7	1	LCK-3
....agaauuguaaggguuuugaga..	1	0	LT-1
....agaauuguaaggguuuugaga..	31	0	LCK-3
....Cgaauuguaaggguuuugaga..	1	1	LT-2
....agaauuguaaggguuuugaga..	9	0	LT-3
....agaauuguaaggguuuugaga..	22	0	LT-2
....agNauuguaaggguuuugaga..	1	1	LT-2
....agaauuguaaggguuuugaga..	2	0	JCK-2
....agaauuguaaggguuuugaga..	2	0	JCK-1
....agaauuguaaggguuuugagag...	2	0	JCK-3
....agaauuguaaggguuuugagag...	4	0	LCK-3
....agaauuguaaggguuuugagaU...	1	1	LT-3
....agaauuguaaggguuuugagag...	1	0	JCK-1
....agaauuguaaggguuuugagag...	2	0	JCK-2
....agaauuguaaggguuuugagag...	3	0	LT-2
....agaauuguaaggguuuugagag...	3	0	LT-1
....agaauuguaaggguuuugaAaga..	10	1	LCK-1
....agaauuguaaggguuuugagaga..	2	0	LT-1
....agaauuguaaggguuuugagaga..	2	0	LT-2
....agaauuguaaggguuuugagaga..	2	0	JT-3
....agaauuguaaggguuuugagaga..	2	0	JCK-1
....agaauuguaaggguuuugaAaga..	12	1	LCK-2
....agaauuguaaggguuuugagaga..	5	0	JCK-2
....agaauuguaaggguuuugagaga..	1	0	JCK-3
....agaauuAuaaggguuuugagaga..	1	1	JCK-1
....agaauuguaaggguuuugagaga..	2	0	LT-3
....agaauuguaaggguuuugagaga..	4	0	JT-2
....agaauuguaaggguuuugagaga..	5	0	LCK-3
....agaauuguaaggguuuugagagaa...	1	0	LT-1
....agaauuguaagAguuuugagagaa...	1	1	JCK-1
....agaauuguaaggguuuugagagaC...	3	1	LCK-3
....agaauuguaagAguuuugagagaa...	1	1	JCK-2
....agaauuguaaggguuuugagagaa...	1	0	JCK-2
....agaauuguaaggguuuugagagaC...	3	1	LT-2
....agaauuguaagAguuuugagagaa...	1	1	JCK-3
....agaauuguaaggguuuugagagaC...	1	1	LT-3
....agaauuguaaggguuuugagagaa...	1	0	LCK-3
....agaauuguaaggguuuugagagaaa..	3	0	JCK-1
....agaauuguaaggguuuugagagaCa..	7	1	LCK-2
....agaauuguaaggguuuugagagaCa..	5	1	LCK-1
....agaauuguaaggguuuugagagaCa..	4	1	LT-1
....agaauuguaaggguuuugagagaCa..	7	1	LCK-3
....agaauuguaaggguuuugagagaCa..	1	1	LT-2
....agaauuguaaggguuuugagagaCauua...	2	1	LCK-3
....agaauuguaaggguuuugagagaCauuauu...	1	1	LCK-3
....gaauuguaaggguuuuga..	23	0	LCK-3
....gaauuguaaggguuuuga..	1	0	LT-1
....gaauuguaaggguuuuga..	3	0	LT-2
....gaauuguaaggguuuugag...	7	0	LT-3
....gaauuguaaggguuuugag...	1	0	JT-2
....gaauuguaaggguuuugag...	1	0	JCK-2
....gaauuguaaggguuuugag...	5	0	LT-1
....gaauuguaaggguuuugag...	5	0	LCK-3
....gaauuguaaggguuuugag...	3	0	JT-3
....gaauuguGaggguuuugaga..	5	1	JCK-2
....Aaauuguaaggguuuugaga..	1	1	LT-1
....gaauuguaaggguuuugaga..	9	0	LT-3
....gaauuguaaggguuuugaga..	2	0	JCK-3
....gaauuguaaggguuuugaga..	12	0	LT-2
....gaauuguaaggguuuugaga..	4	0	LT-1
....gaauuguaaggguuuugaga..	6	0	JT-3
....gaauuguaagggCuuugagag...	2	1	LCK-3
....gaauuguaaggguuuugagag...	2	0	LT-2
....gaauuguaaggguuuugagag...	3	0	LT-3
....gaauuguaaggguuuugagag...	2	0	JCK-3

Star Mature

uacauuuuaauuauauauuugggguauuuauuuauuuauuuguuguuuguuaaauuauuuggguuccuugguuuucgagaaguacgaauaucagguuuugugaaaagguuuuaaaaggggaacuuuuccaaccgagagaauuguaaggguuuugagagaaauuauuauuuccaauuaauuauuaucuaucugcuuauuaccgugguauuauauuguauaguagauaggucacucacugagaugauuagcau

gaauuguaaggguuuugagag	2	0	JCK-1
gaauuguaaggguuuugagag	3	0	JCK-2
gaauuguaaggguuuugagaga	6	0	LT-3
gaauuguaaggguuuugagagG	1	1	JCK-2
gaauuguaaggguuuugagaga	1	0	JCK-1
gaauuguaaggguuuugagaga	2	0	LT-1
gaauuguaaggguuuugagaga	3	0	JCK-2
gaauuguaaggguuuugagagG	1	1	JCK-3
gaauuguaaggguuuugagaga	3	0	JCK-3
gaauuguaaggguuuugagaga	6	0	LCK-3
gaauuguaaggguuuugagagaC	1	1	LT-1
gaauuguaaggguuuugagagaa	1	0	JCK-1
gaauuguaaggguuuugagagaa	10	0	JCK-2
gaauuguaaggguuuugagagaa	2	0	LT-3
gaauuguaaggguuuugagagaa	1	0	JCK-3
gaauuguaaggguuuugagagaa	3	0	LT-2
gaauuguCaggguuuugagagaa	1	1	JCK-2
gaauuguaaggguuuugagagaaa	2	0	JCK-3
gaauuguaaggguuuugagagaCa	1	1	LT-2
gaauuguaaggguuuugagagaaa	1	0	LCK-3
gaauuguaaggguuuugagagaaa	1	0	JCK-2
gCauuguaaggguuuugagagaaa	1	1	LCK-3
gaauuguaaggguuuugagagaCa	1	1	LT-1
aauuguaaggguuuugag	6	0	LCK-3
aauuguaGggguuuugag	1	1	LCK-3
aauuguaaggguuuugag	3	0	JCK-3
aauuguaaggguuuugag	1	0	JCK-1
aauuguaaggguuuugag	2	0	JCK-2
aauuguaaggguuuugag	10	0	LT-2
aauuguaaggguuuugaga	22	0	LCK-3
aauuguaagAguuuugaga	1	1	LT-3
aauuguaaggguuuugaga	4	0	LT-1
aauuguaaggguuGugaga	1	1	LT-3
aauuguaaggguuuugaga	1	0	LT-2
aauuguaaggguuuugaga	3	0	LT-3
aauuguaaggguuuugagag	8	0	LCK-3
aauuguaaggguuuugagag	1	0	LT-3
aauuguaaggguuuugagag	3	0	LT-2
aauuguaaggguuuugagaga	5	0	LT-2
Cauuguaaggguuuugagaga	1	1	LT-2
aauuguaaggguuuugagaga	2	0	LT-3
aauuguaaggguuuugagaga	2	0	LT-1
aauuguaaggguuuugagagaC	3	1	LT-2
aauuguaaggguuuugagagaCa	21	1	LCK-1
aauuguaaggguuuugagagaaa	3	0	LCK-2
aauuguaaggguuuugagagaCa	2	1	JCK-3
aauuguaaggguuuugagagaCa	2	1	LT-1
aauuguaaggguuuugagagaCa	1	1	LT-3
aauuguaaggguuuugagagaaa	7	0	LCK-1
aauuguaaggguuuugagagaCa	8	1	LT-2
aauuguaaggguuuugagagaCa	18	1	LCK-2
auuguaaggguuuugaga	25	0	LCK-2
auuguaaggguuuugaga	20	0	LCK-3
auuguaaggguuuugaga	7	0	LT-2
auuguaaggguuuugaga	18	0	LCK-1
auuguaaggguuuugaga	11	0	JT-3
auuguaaggguuuugaga	4	0	LT-3
auuguaaggguuuugaga	4	0	LT-1
auuguaaggguuuugaga	5	0	JT-2
auuguaaggguuuugaAa	1	1	LT-3
auuguaaggguuuugagag	1	0	LT-1
auuguaaggguuuugagag	1	0	JCK-3
auuguaagAguuuugagag	1	1	LCK-1
auuguaaggguuuugagag	31	0	LCK-2
auuguaaggguuuugagag	30	0	LCK-1
auuguCaggguuuugagag	1	1	LCK-2
auuguaaggguuuugagaga	1	0	LT-1
auuguaaggguuuugagaga	7	0	LT-2
auuguaaggguuGugagaga	1	1	LT-3
auuguaaggguuuugagaga	2	0	JCK-3

Star **Mature**

uacauuuuaauuauauauuugggguauuuauuuauuuauuuguuguuuguuaaauuauuugguuccuugguuuucgagaaguacgaauaucagguuuugugaaaagguuuuaaaagggaacuuuuccaaccgagagaauuguaaggguuuugagagaaauuauuauuuccaauuaauuauuaucuaucugcuuauuaccgugguauuauauuguauaguagauaggucacucacugagaugauuagcau

auuguaaggguuuugagaga	1	0	LT-3
auuguaaggguuuugagagaa	2	0	LT-2
auuguaaggguuuugagagaC	1	1	LT-3
auuguaaggguuuugagagaa	1	0	LT-3
auuguaaggguuuugagagaCa	8	1	LCK-3
auuguaaggguuuugagagaCa	3	1	LT-2
auuguaaggguuuugagagaCa	3	1	LT-3
auuguaaggguuuugagagaCa	1	1	LT-1
auuguaaggguuuugagagaaauu	1	0	JCK-2
uuguaaggguuuugagag	8	0	LCK-3
uuguaaggguuuugagaga	3	0	LT-2
uuguaaggguuuugagagaa	2	0	LT-3
uuguaagggCuuugagagaa	1	1	LT-1
uuguaagAguuuugagagaaa	2	1	JCK-2
uuguaaggguuuugagagaaa	1	0	LT-2
uuguaaggguuuugagagaCa	1	1	LT-3
uuguaaggguuuugagagaaauu	1	0	JCK-1
uuguaaggguuuugagagaaauu	3	0	JCK-2
uguaaggguuuugagaga	5	0	LT-1
Aguaaggguuuugagaga	1	1	JCK-3
uguaaggguuuugagaga	2	0	LT-3
uguaaggguuuugagaga	5	0	LCK-3
uguaaggguuuugagagaa	6	0	LCK-3
uguaaggguuuugagagaC	8	1	LCK-3
uguaaggguuuugagagaCa	1	1	JCK-3
uguaaggguuuugagagaCau	2	1	JCK-2
uguaaggguuuugagagaaau	1	0	JCK-2
uguaaggguuuugagagaaau	1	0	JCK-3
uguaaggguuuugagagaCau	1	1	JCK-1
uguaaggguuuugagagaaauuG	1	1	JCK-3
uguaaggguuuugagagaaauuG	3	1	JCK-2
uguaaggguuuugagagaaauuG	3	1	JCK-1
guaaggguuuugagagaaauu	1	0	LT-2
uaaggguuuugagagaCa	3	1	LCK-3
uaaggguuuugagagaaa	1	0	LT-2
aaggguCuugagagaaauuauua	1	1	JCK-1
aaggguCuugagagaaauuauua	2	1	JCK-3
aaggguuuugagagaCauuauuau	1	1	JCK-1
agggCuuugagagaaauuauuauu	2	1	LCK-3
cuuauuacUgugguauuauauugu	1	1	JCK-3
cuuauuacUgugguauuauauugu	2	1	JCK-1
accgugguauuauauugua	1	0	LT-2
gugguauuGuauuguauagu	1	1	LT-1
auuauauuguauaguaga	1	0	LT-1
auuauauuguauaguagauaggGc	1	1	LT-1
auuAuauaguagauaggu	1	1	LCK-2
auuguaCaguagauaggu	1	1	LT-2
auuguauaguagauaggu	30	0	LCK-1
auuguauaguagauaggu	18	0	LCK-2
uaggucacuAacugagaug	12	1	LCK-3
ucacucacugaAaugauuagc	1	1	LT-3
cacucacugagauAauuagc	1	1	LT-3
acucacugagaugauuagcau	1	0	LT-1
cucacugagaugauuagc	1	0	LT-2
cucacugagaugaCuagc	1	1	JCK-1
cucacugagaugaCuagc	1	1	JCK-2
cucacugagaugauuagc	1	0	LT-1
cucacugagaugauuagc	1	0	LT-3
cucacugagaugauuagca	1	0	LT-1
